# Impact of Histone H4 Lysine 20 Methylation on 53BP1 Responses to Chromosomal Double Strand Breaks

**DOI:** 10.1371/journal.pone.0049211

**Published:** 2012-11-28

**Authors:** Andrea J. Hartlerode, Yinghua Guan, Anbazhagan Rajendran, Kiyoe Ura, Gunnar Schotta, Anyong Xie, Jagesh V. Shah, Ralph Scully

**Affiliations:** 1 Department of Medicine, Harvard Medical School and Beth Israel Deaconess Medical Center, Boston, Massachusetts, United States of America; 2 Department of Systems Biology, Harvard Medical School and Renal Division, Brigham and Women's Hospital, Boston, Massachusetts, United States of America; 3 Division of Gene Therapy Science, Osaka University School of Medicine, Osaka, Japan; 4 Ludwig Maximilians University and Munich Center for Integrated Protein Science (CiPSM), Adolf-Butenandt-Institute, Munich, Germany; The University of Hong Kong, Hong Kong

## Abstract

Recruitment of 53BP1 to chromatin flanking double strand breaks (DSBs) requires γH2AX/MDC1/RNF8-dependent ubiquitination of chromatin and interaction of 53BP1 with histone H4 methylated on lysine 20 (H4K20me). Several histone methyltransferases have been implicated in 53BP1 recruitment, but their quantitative contributions to the 53BP1 response are unclear. We have developed a multi-photon laser (MPL) system to target DSBs to subfemtoliter nuclear volumes and used this to mathematically model DSB response kinetics of MDC1 and of 53BP1. In contrast to MDC1, which revealed first order kinetics, the 53BP1 MPL-DSB response is best fitted by a Gompertz growth function. The 53BP1 MPL response shows the expected dependency on MDC1 and RNF8. We determined the impact of altered H4K20 methylation on 53BP1 MPL response kinetics in mouse embryonic fibroblasts (MEFs) lacking key H4K20 histone methyltransferases. This revealed no major requirement for the known H4K20 dimethylases Suv4-20h1 and Suv4-20h2 in 53BP1 recruitment or DSB repair function, but a key role for the H4K20 monomethylase, PR-SET7. The histone methyltransferase MMSET/WHSC1 has recently been implicated in 53BP1 DSB recruitment. We found that *WHSC1* homozygous mutant MEFs reveal an alteration in balance of H4K20 methylation patterns; however, 53BP1 DSB responses in these cells appear normal.

## Introduction

Double-strand breaks (DSBs) trigger a complex set of cellular responses, the concerted action of which ensures appropriate repair and suppresses genomic instability [1], [2]. Defective DSB responses can cause immune deficiency, increased cancer predisposition and premature aging in mammals [3], [4], [5]. Phosphorylation of the variant histone H2AX to form γH2AX″ is an early DSB response that marks megabases of chromatin flanking a mammalian DSB [6], [7]. γH2AX recruits MDC1 by directly binding its tandem BRCT repeat and MDC1, in turn, recruits Mre11/Rad50/NBS1/Atm and the E3 ubiquitin ligase, RNF8 to chromatin [8], [9], [10], [11], [12], [13]. RNF8 activates the downstream E3 ubiquitin ligase RNF168 and mediates ubiquitination of chromatin components [14], [15]. This ubiquitination cascade is required for the recruitment of BRCA1/BARD1/Abraxas/Rap80 and of 53BP1 to γH2AX chromatin [16].

H2AX, MDC1 and 53BP1 contribute to *IgH* class-switch recombination (CSR) [17] and the fusion of dysfunctional telomeres [18] – both specialized examples of non-homologous end-joining (NHEJ). For DSBs encountered in a non-specialized context, γH2AX/MDC1 regulates homologous recombination (HR) between sister chromatids [19], [20], whereas 53BP1 contributes primarily to NHEJ, revealed by a “hyperrecombination” phenotype when 53BP1 is inhibited [19], [21]. 53BP1 DSB repair functions are at least in part independent of *H2AX*, as revealed by the more severe CSR defect in *53BP1* ^−/−^ compared to *H2AX* ^−/−^ mice, the persistence of 53BP1-mediated inhibition of “hyperrecombination” in *H2AX* ^−/−^ cells, and the transient accumulation of 53BP1 at DSBs in *H2AX* ^−/−^ cells [19], [22], [23].

Recruitment of 53BP1 to γH2AX chromatin is mediated by direct interaction between the 53BP1 tandem Tudor repeat and histone H4 methylated on lysine 20 (H4K20me) [24]. An earlier study reported that 53BP1 can also be recruited to sites of DSBs by interaction with dimethylated lysine 79 of histone H3 [25]. However, another report showed that loss of histone H3 K79 dimethylation does not affect 53BP1 recruitment to IR-induced foci (Botuyan, 2006).The 53BP1 tandem Tudor repeat exhibits similar binding affinities for H4K20me2 and me1 in vitro but no specific binding to H4K20me0, H4K20me3 or H3K79me2 [24], [26].

Methylation of histone H4K20 requires PR-Set7/SET8, which monomethylates H4K20 and is dynamically regulated during the cell cycle [27], [28], [29]. Histone H4K20me1 is a substrate of Suv4-20h1 and Suv4-20h2, which convert H4K20me1 primarily to H4K20me2 (and to H4K20me3 in heterochromatin) [30], [31]. *PR-Set7* ^−/−^ mice reveal early embryonic lethality accompanied by defective chromatin condensation and chromosome fragility [28]. *Suv4-20h1* ^−/−^/*Suv4-20h2* ^−/−^ (here termed “*Suv4-20h1/2* null”) mice are live-born but die perinatally, exhibiting mild chromosome fragility and impaired CSR [30]. In *Suv4-20h1/2* null MEFs, H4K20me2 and H4K20me3 marks are erased and H4K20me1 is the predominant H4K20me species [30]. In these cells, loss of the H4K20me2 mark was found to slightly delay 53BP1 focus formation [30].

In wild type primary mouse embryonic fibroblasts (MEFs), ∼90% of all histone H4 molecules are dimethylated at K20 [30]. Despite this, 53BP1 chromatin accumulation near DSBs is restricted to γH2AX -marked chromatin. This led to the proposal that the H4K20me2 mark may be buried in the context of higher order chromatin structure and be exposed by localized chromatin decondensation triggered, in mammals, by γH2AX/MDC1/RNF8 (reviewed in [16]). Alternative models posit more specific alterations in chromatin at the DSB. In this regard, PR-Set7 is detectable at DSBs in the G2 phase of the cell cycle [32] and the histone methyltransferase MMSET/WHSC1 was proposed to contribute to 53BP1 chromatin recruitment at DSBs by catalyzing the local deposition of H4K20me2 near the DSB [33]. These recent reports raise questions regarding the quantitative contribution of distinct histone methyltransferases to the 53BP1 DSB response. We approached this by studying 53BP1 DSB repair function and DSB recruitment in MEFs lacking specific histone methyltransferases. To quantify 53BP1 DSB response kinetics, we developed a multi-photon laser (MPL) system to target DSBs to subfemtoliter volumes of the nucleus, accompanied by real time imaging of 53BP1 DSB recruitment.

## Results

### 53BP1 DSB repair function in *Suv4-20h1/2* null MEFs

**Figure 1:**
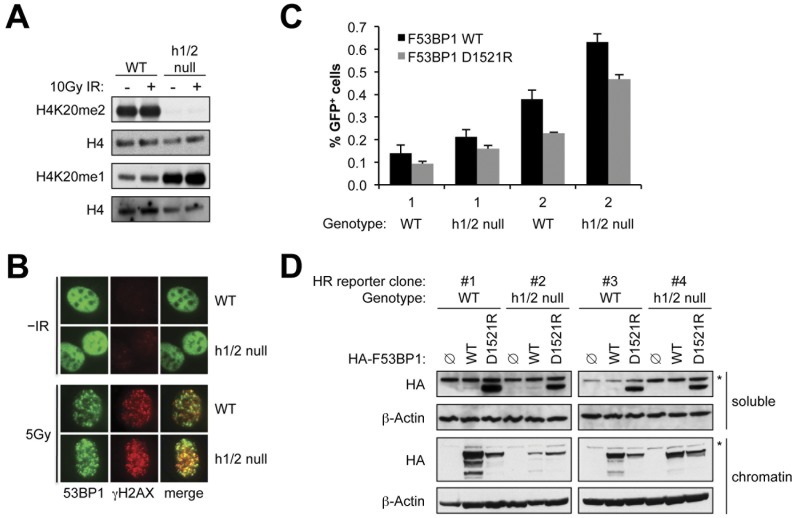
Histone H4K20me2 mark is dispensable for 53BP1-mediated DSB repair. **A**) Immunoblotting for H4K20me2, H4K20me1, and H4 (loading control) in individually derived wild type (WT) and *Suv4-20h1/2* null (h1/2 null) cell lines that received no treatment or 10 Gy of IR. **B**) 53BP1 and γH2AX focus formation 10 minutes after 5 Gy IR treatment of wild type and *Suv4-20h1/2* null cells. **C**) I-SceI-induced HR frequencies (indicated by GFP^+^ products) in two independent wild type and *Suv4-20h1/2* null HR reporter cell lines transiently transfected with HA-F53BP1 WT or the HA-F53BP1 D1521R mutant expression plasmids. Bars represent mean of triplicate samples. Error bars indicate s.e.m.. t test of F-53BP1 WT vs. D1521R: Clone 1 WT: not significant (NS); Clone 1 h1/2 null: NS;Clone 2 WT p = 0.025; Clone 2 h1/2 null: p = 0.027. **D**) Levels of transiently expressed HA-tagged F53BP1 proteins and β-actin loading control corresponding to the experiment in **C** in both the soluble and chromatin fractions. Note tight chromatin association of wt F53BP1 fragment and abundant soluble fraction of F53BP1 D1521R protein. *: background band.

To study the impact of altered chromatin-wide H4K20 methylation on 53BP1 DSB repair functions, we introduced I-SceI-inducible HR reporters [34] into *Suv4-20h1/2* null MEFs and, in parallel, MEFs from wild type littermates. We generated clones that carry only one randomly integrated, intact copy of the HR reporter (see Materials and Methods) and studied two independent clones of each genotype. The background level of GFP^+^ products in the absence of I-SceI was <0.01% for all clones. As reported previously [30], *Suv4-20h1/2* null MEFs revealed no H4K20me2 signal and a compensatory increase in H4K20me1 (**Figure 1A**). Exposure of these cells to 10 Gy ionizing radiation (IR) did not alter the abundance of these marks in either cell type, but both wild type and *Suv4-20h1/2* null MEFs revealed robust recruitment of 53BP1 to γH2AX chromatin in response to IR (5 Gy; **Figure 1B**).

To inhibit 53BP1, we used a previously characterized fragment of 53BP1 (“F53BP1”, corresponding to residues 1221–1714, comprising key chromatin localization domains of 53BP1 including the tandem Tudor repeat and an oligomerization domain) that, when overexpressed, interferes with endogenous 53BP1 DSB repair functions [19]. As described previously, transient overexpression of F53BP1 stimulates I-SceI-mediated HR, in comparison with a functionally null mutant F53BP1 D1521R, which lacks the ability to interact with H4K20me species and fails to localize IR-induced nuclear foci [19]. As described previously, expression of F53BP1 D1521R has no impact on I-SceI-induced HR in comparison with transfected empty vector, and F53BP1 fails to stimulate HR in *53BP1* ^−/−^ cells, indicating a specific interference with 53BP1 DSB repair function [19]. Transfection of *Suv4-20h1/2* null or wild type HR reporter MEF clones with F53BP1 stimulated HR to a similar extent, in comparison to F53BP1 D1521R (**Figure 1C**). As expected, wild type F53BP1 was more strongly associated with the chromatin fraction than the F53BP1 D1521R fragment (**Figure 1D**). These results show that chromatin-wide loss of the H4K20me2 mark does not abolish the recombination-suppression function of 53BP1.

### Altered 53BP1 localization following depletion of H4K20me1

**Figure 2:**
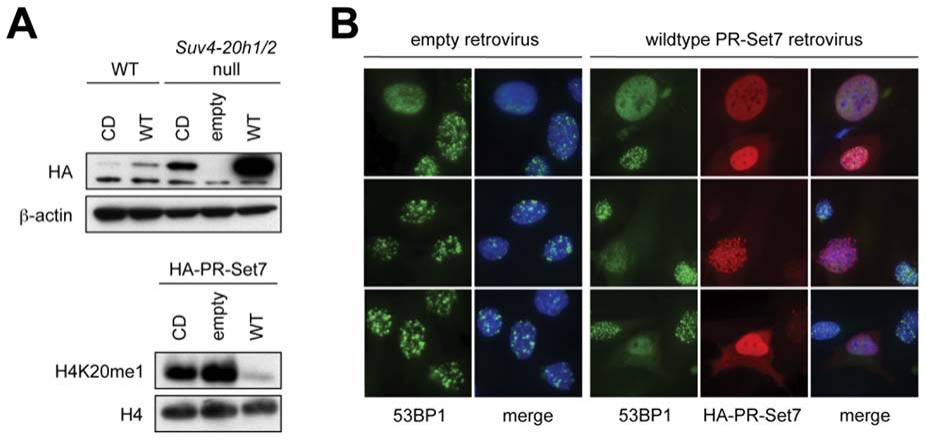
Perturbed 53BP1 focus formation in cells depleted of H4K20me1. **A**) Top: Immunoblot for HA-PR-Set7 proteins (WT = wild type, CD = catalytically dead) and β-actin loading control in wild type and *Suv4-20h1/2* null cells. Bottom: Immunoblot for H4K20me1 and H4 loading control in *Suv4-20h1/2* null cells infected with empty, PR-Set7 WT and PR-Set7 CD retrovirus. **B**) 53BP1 focus formation and HA immunofluorescence staining in *Suv4-20h1/2* null cells expressing empty or HA-PR-Set7 WT retrovirus, 30 minutes after 3 Gy IR treatment.

To determine whether the H4K20me1 mark is necessary for 53BP1 function, we used HA-tagged PR-Set7 overexpression to inhibit the endogenous PR-Set7 enzyme [26]. Following retroviral transduction of wild type or *Suv4-20h1/2* null MEFs with wild type or catalytically inactive PR-Set7, with selection of transduced pools of cells in puromycin, we observed high levels of ectopic PR-Set7 in *Suv4-20h1/2* null MEFs expressing wild type PR-Set7 and cells overexpressing the wild type enzyme revealed significant depletion of the H4K20me1 mark (**Figure 2A**). We do not understand why wild type but not mutant PR-Set7 had this effect; this could be a reflection of the greater abundance of the wild type protein, the reasons for which are not clear. (Each *PR-Set7* cDNA construct was resequenced and confirmed to be correct.) As described previously, depletion of the H4K20me1 mark caused progressive growth impairment, leading ultimately to cell cycle arrest and limiting the viability of the culture to approximately one week [26]. This progressive cell cycle arrest made measurement of HR functions in these cultures impractical. IR-induced 53BP1 focus formation appeared to be altered in HA-PR-Set7-expressing cells (**Figures 2B**). However, a large variety of different IR-induced 53BP1 nuclear patterns was noted, consistent with reports of DNA damage caused by depletion of PR-Set7 [26], [35]. This made accurate quantitation of 53BP1 focus formation problematic.

### A multi-photon laser system for kinetic analysis of 53BP1 chromatin responses to chromosomal DSBs

**Figure 3:**
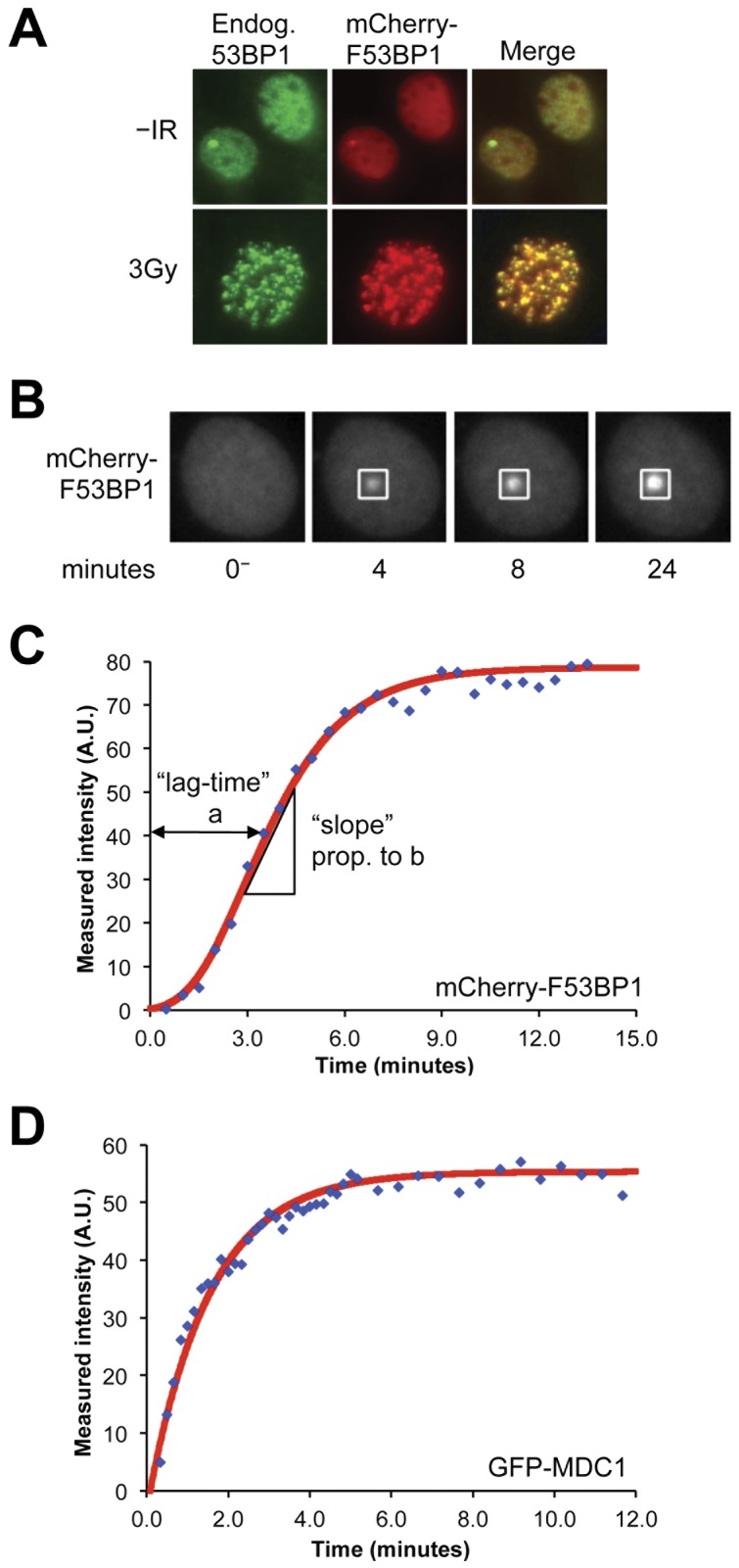
Recruitment of mCherry-F53BP1 to sites of damage induced by multi-photon laser. **A**) Endogenous 53BP1 and mCherry-F53BP1 focus formation in wild type MEFs 30 minutes after 3 Gy IR treatment. **B**) Representative images of mCherry-F53BP1 accumulation over time at site of MPL-induced DNA damage. **C**) Plot of accumulation kinetics of mCherry-F53BP1 to MPL-induced damage demonstrates the lag in 53BP1 recruitment. Blue diamonds represent raw data, red line represents mathematical (Gompertz) model fit to raw data. The mathematical model allows the parameterization of different kinetic behaviors. The “lag-time” is the time it takes for protein recruitment to reach the inflection point of the curve, where the rate of change of the slope is equal to 0. The “slope” of the curve is measured at the inflection point. **D**) Plot of the accumulation of GFP-MDC1 to MPL-induced damage over time is well fitted by first order kinetics (red line).

To quantify 53BP1 DSB response kinetics more directly, we adapted a multi-photon laser (MPL) system [36], [37], [38], [39] for targeting DSBs to defined, subfemtoliter volumes of the nucleus and combined this with real-time imaging of the 53BP1 response. As a marker of 53BP1, we fused mCherry to the minimal 53BP1 localization domain, F53BP1, and expressed this stably at low levels in wild type MEFs following retroviral transduction and subsequent puromycin selection. Consistent with previous studies, mCherry-F53BP1 colocalized perfectly with endogenous 53BP1 in response to IR [18] (**Figure 3A**). In contrast, the F53BP1 D1521R mutant fails to accumulate at DSBs. MPL-mediated damage was achieved by focusing the collimated light of a near infrared femtosecond laser source, tuned to 780 nm, through a high numerical aperture objective. The femtosecond source results in high peak intensities in the diffraction-limited focus and subsequent multi-photon absorption in the local environment. The mechanism of DSB-induction by MPL is not known; however, the generation of reactive oxygen species is thought to be a major contributor to DNA damage at the site of laser damage, one product of which will be the generation of chromosomal DSBs [40].

In response to MPL-induced damage, we observed robust recruitment of mCherry-F53BP1 to MPL lesions (**Figures 3B and 3C**). By titrating the average power of the laser, we identified a threshold below which 53BP1 focus formation became unreliable (**Table S1**). Pre-incubation of cells with the sensitizing agents BrdU (**Table S1**) and/or Hoechst dye reduced this threshold. We selected an average laser power of 25 mW for all subsequent experiments and elected to avoid pre-incubation with DNA sensitizing agents. At this dose, >90% of MPL lesions triggered a 53BP1 response in wild type MEFs. We quantified the kinetics of 53BP1 recruitment to MPL-induced damage as described in Materials and Methods. Consistent with a previous study using a different laser method for the induction of DSBs, we noted that 53BP1 recruitment did not fit first-order kinetics [41]. Indeed, there was a consistent time lag in 53BP1 accumulation with respect to the laser pulse, indicated by an inflection point in the F-53BP1 accumulation time-course at ∼2–4 minutes (**Figure 3C**). Symmetrical sigmoid functions, or two-step first-order kinetic models were unable to model F53BP1 recruitment kinetics. In contrast, a double exponential Gompertz function [42] gave a reliable fit to the observed kinetics; we used an iterative non-linear curve fitting algorithm (MATLAB) to obtain Gompertz parameters that best fit the observed 53BP1 recruitment time-course for each individual cell imaged (for example, **Figure 3C**), according to the Gompertz growth equation:
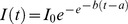
where *I(t)* is the intensity at time *t*, *I_0_* the final focus intensity, *a* (here termed “lag-time”) is the time taken to reach the inflection point and *b* (here termed “slope”) is proportional to the gradient of the curve at the inflection point, corrected for final focus intensity. Final focus intensity was influenced, in part, by the abundance of the mCherry-F53BP1 protein in an individual cell. To control for this source of variation, we calculated the “intensity ratio” as the fractional increase of intensity of the focus, compared to the background nuclear fluorescence:
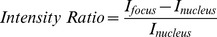
where *I_focus_* is the peak focus intensity and *I_nucleus_* is the background nuclear fluorescence intensity at the same time point (see Materials and Methods). For example, if the focus fluorescence intensity were twice that of background, the intensity ratio would be 1. If there were no focus formation, the intensity ratio would be zero.

53BP1 can accumulate transiently at DSBs in the absence of *H2AX* or *MDC1* [23], [41]. Indeed, we noted 53BP1 recruitment to MPL-induced DSBs in a fraction of *MDC1* ^−/−^ MEFs (data not shown); however, the peak intensity observed in *MDC1* ^−/−^ MEFs was much lower than that noted in wild type cells. This shows that the major 53BP1 signal observed in cells with intact H2AX and MDC1 corresponds to the *H2AX/MDC1*-dependent 53BP1 chromatin response.

### RNF8 controls a slow step in 53BP1 chromatin recruitment

**Figure 4:**
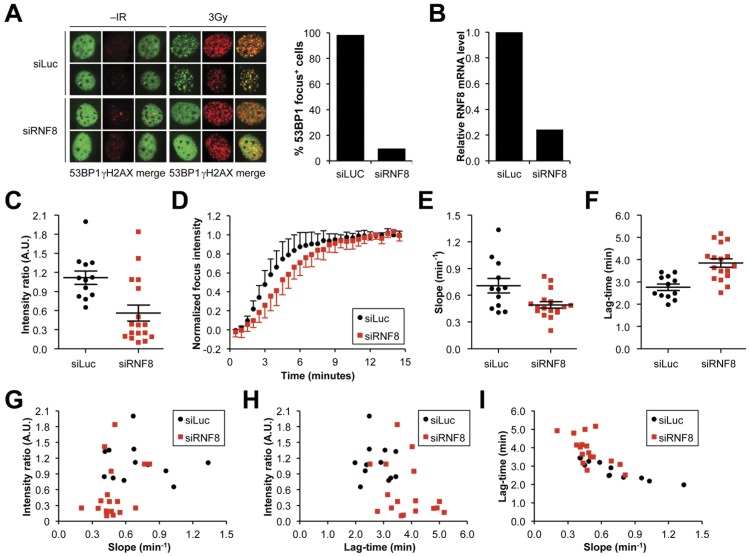
Quantitative impairment in F53BP1 MPL responses in cells depleted of RNF8. **A**) Endogenous 53BP1 and γH2AX focus formation in wild type MEFs transfected with control siRNA or siRNA directed against RNF8 1 hour after 3 Gy IR treatment. Quantification of the proportion of cells containing endogenous 53BP1 foci in control siRNA and siRNF8 treated cells. **B**) Quantification by RT-qPCR of the relative RNF8 mRNA level in cells transfected with control siRNA or siRNF8. **C**) Plot of peak fluorescence intensity for each responding cell. Bar represents the mean peak fluorescence intensity for each data set and error bars indicate SEM (p<0.004). Black circles: siLuc; Red squares: siRNF8. **D**) Plot of averaged mCherry-F53BP1 fluorescence accumulation over time normalized to a peak fluorescence intensity of 1.0 for each responding cell. **E**) Plot of slope of fluorescence accumulation at the inflection point for each responding cell. Bar represents the mean slope in fluorescence accumulation at the inflection point for each data set and error bars indicate SEM (p<0.013). **F**) Plot of lag-time in fluorescence accumulation for each responding cell. Bar represents the mean lag-time in fluorescence accumulation for each data set and error bars indicate SEM (p<0.0004). **G**) Plot of slope *vs*. peak fluorescence intensity for each responding cell. **H**) Plot of lag-time *vs*.peak fluorescence intensity for each responding cell. **I**) Plot of slope *vs*. lag-time for each responding cell.

The Gompertz-type recruitment kinetics noted in 53BP1 accumulation following MPL-induced damage could reflect cooperative binding interactions between 53BP1 molecules. We cannot rule out such cooperative interactions, given that F53BP1 contains a dimerization/oligomerization domain, which is required for its chromatin localization [43]. However, we noted recruitment kinetics of mCherry-F53BP1 in *53BP1* ^−/−^ MEFs that were quantitatively similar to wild type MEFs (data not shown), suggesting that only sequences contained within F53BP1 are required to reproduce the Gompertz-type pattern of recruitment. A second possible explanation for the complex early time course of 53BP1 recruitment could be that the formation of DSBs following MPL exposure is itself delayed. We therefore studied MPL response kinetics of MDC1, and observed robust accumulation of GFP-tagged MDC1 at sites of MPL-induced damage. Strikingly, GFP-MDC1 recruitment revealed rapid first-order kinetics (**Figure 3D** **; Movie S1**). This suggests that the steps of the DSB response leading up to MDC1 recruitment are rapid, whereas a slower process is interposed between MDC1 and 53BP1 recruitment. This step entails a ubiquitination cascade controlled by RNF8 [11], [12], [13]. To determine the impact of RNF8 dysfunction on 53BP1 recruitment kinetics, we used siRNAs to deplete RNF8 (“siRNF8”), *versus* control siRNA that targets luciferase (“siLuc”). Consistent with previous work, siRNF8 strongly suppressed IR-induced 53BP1 focus formation but left γH2AX focus formation intact [11], [12], [13] (**Figures 4A and 4B**). Similarly, siRNF8 perturbed mCherry-F53BP1 accumulation at MPL-induced lesions in comparison with siLuc, as revealed by altered distributions of each Gompertz kinetic parameter (**Movies S2** and S**3**). The mean intensity ratio of the MPL-induced 53BP1 focus was reduced (**Figure 4C**), the mean lag time was increased and the mean slope decreased (**Figures 4D**–**4F**). Thus, as with the 53BP1 response to IR-induced DSBs, efficient recruitment of 53BP1 to MPL-induced DSBs requires an intact MDC1/RNF8 pathway. The persistence of a 53BP1 MPL response in RNF8-depleted cells might appear to be inconsistent with the loss of IR-induced 53BP1 foci in the same setting. However, this is likely the result of differences in the detection threshold of the 53BP1 signal; for IR-induced DSBs, each focus corresponds to one DSB, whereas MPL-induced foci represent the response to multiple clustered DSBs. This clustering is likely to increase the sensitivity of detection, hence resulting in the ability to detect residual 53BP1 signals of diminished intensity that would be undetectable in the response to IR.

We analyzed the relationship between each pair of kinetic parameters in the Gompertz model. This revealed no correlation between peak intensity and slope and a weak negative correlation between peak intensity and lag-time (**Figures 4G and 4H**). In contrast, lag-time and slope were strongly negatively correlated (**Figure 4I**). Consistent with this, a plot of lag-time vs. 1/slope revealed a strong linear relationship (**Figure S1**).

### Impact of H4K20 methylation status on 53BP1 recruitment to MPL-induced DSBs

**Figure 5:**
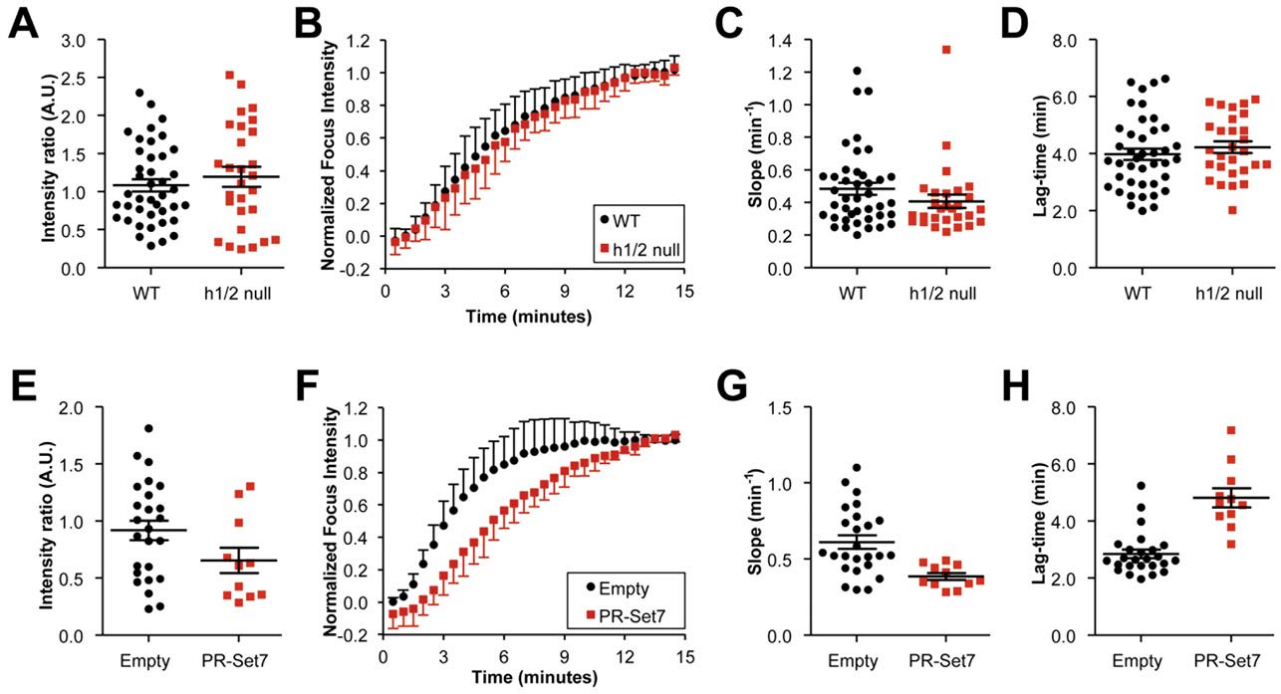
Impact of altered H4K20 methylation on recruitment kinetics of F53BP1 to MPL-induced DSBs. **A**)–**D**) Comparison of wild type (WT) vs. *Suv4-20h1/2* null (h1/2 null) MEFs. **E**)–**H**) Comparison of *Suv4-20h1/2* null MEFs expressing empty retrovirus vs. PR-SET7 retrovirus. **A**) and **E**) Plot of peak fluorescence intensity for each responding cell. Note that the high proportion of non-responsive cells in PR-SET7 cultures are not represented in this analysis. Bar represents the mean peak fluorescence intensity for each data set and error bars indicate s.e.m. (**A**: p<0.45; **E**: p<0.09). **B**) and **F**) Plot of averaged mCherry-F53BP1 fluorescence accumulation over time, normalized to a peak fluorescence intensity of 1.0 for each responding cell. **C**) and **G**) Plot of slope in fluorescence accumulation at the inflection point for each cell imaged. Bar represents the mean slope and error bars indicate s.e.m. (**C**: p<0.18; **G**: p<0.0026). **D**) and **H**) Plot of lag-time in fluorescence accumulation for each cell imaged. Bar represents the mean lag-time and error bars indicate s.e.m. (**D**: p<0.4; **H**: p<0.0001).

To examine the impact of genome-wide conversion of H4K20me2 to me1 on 53BP1 DSB response kinetics, we studied mCherry-F53BP1 responses to MPL-induced breaks in *Suv4-20h1/2* null MEFs vs. isogenic wild type control MEFs. 53BP1 responses were virtually indistinguishable between the two groups (**Figure 5A–D**; **Movie S4**). Interestingly, the plot of lag-time vs. 1/slope (**Figure S1**) revealed a greater scatter in *Suv4-20h1/2* null MEFs, perhaps suggesting that the early recruitment of 53BP1 is somewhat disorganized in these cells, as was suggested previously [30]. To determine the impact of genome-wide depletion of the H4K20me1 mark on 53BP1 recruitment kinetics, we studied 53BP1 responses to MPL damage in *Suv4-20h1/2* null MEFs stably expressing PR-Set7 (**Figure 2**) vs. control cultures that received empty retrovirus. Strikingly, in PR-Set7-expressing cultures, 9/20 (45%) cells examined revealed no response to MPL-induced breaks (example shown in **Movie S5**), whereas only 3/28 (10.7%) cells in control cultures failed to respond. When *only the responder cells* from each group were analyzed, cultures transduced with PR-Set7 revealed reduced intensity ratio and delayed 53BP1 kinetics, as revealed by increased lag-time and reduced slope values (**Figure 5E–H**; **Movie S6**). Note that **Figure 5** underestimates the impact of PR-SET7 on 53BP1 DSB responses, since the ∼45% of PR-SET7-expressing cells that were non-responsive to MPL were excluded from kinetic analysis. In contrast, PR-SET7 expression did not suppress the accumulation of GFP-MDC1 at MPL lesions (**Figure S2**). These experiments show that overexpression of PR-SET7 and the accompanying depletion of the H4K20me1 mark profoundly and specifically impairs 53BP1 recruitment to γH2AX chromatin.

**Figure 6:**
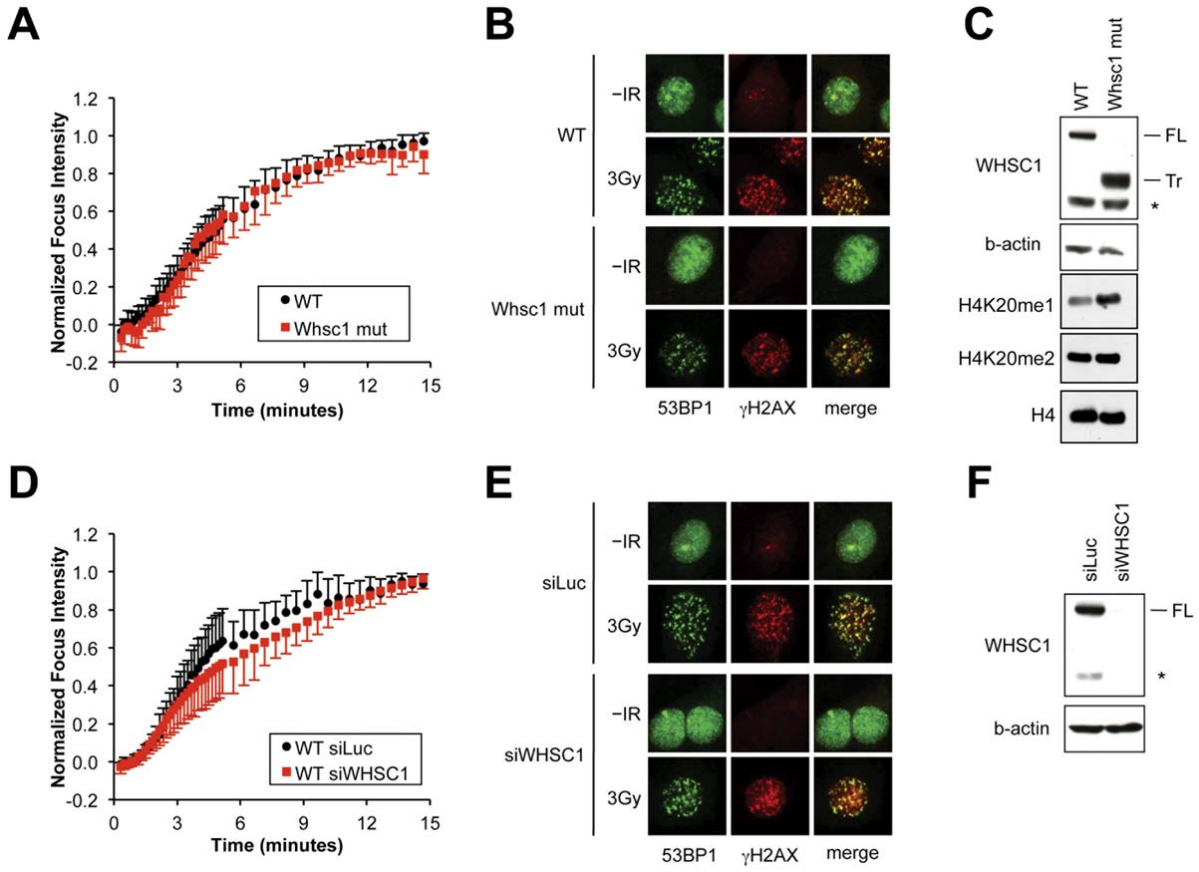
Impact of WHSC1 inactivation on 53BP1 DSB responses. **A**)–**C**). Comparison of wild type (WT) vs. *WHSC1^mut/mut^* (Whsc1 mut) MEFs. **D**)–**F**) Comparison of wild type MEFs transfected with siRNA to Luciferase (siLuc; control sample) or WHSC1 (siWHSC1). **A**) and **D**) Plot of averaged mCherry-F53BP1 fluorescence accumulation over time normalized to a peak fluorescence intensity of 1.0 for each responding cell. Error bars indicate s.d.. **B**) and **E**) Endogenous 53BP1 and γH2AX focus formation in cultures 30 minutes following treatment with 3 Gy IR. **C**) and **F**) Immunoblot with antibody specific for WHSC1 N-terminus and for β-actin (loading control) in test cultures. FL = full length WHSC1, Tr = likely truncated *WHSC1* gene product, * = unidentified band, a potential *MHSC1* gene product in view of its depletion by siWHSC1. Panel **C** also shows abundance of mono- and di-methylated H4K20 in WT vs. *WHSC1* ^mut/mut^ MEFs, with histone H4 as a loading control. Note slightly increased abundance of H4K20me1 in *WHSC1* ^mut/mut^ MEFs.

Recent work has identified a role for the histone H3 lysine 36 methyltransferase, MMSET/WHSC1, in promoting 53BP1 recruitment to nuclear foci in the DNA damage response [33], [44]. Pei et al. reported that MMSET/WHSC1 supports 53BP1 to IR-induced DSBs in human osteosarcoma cells [33]. We studied the role of WHSC1 in the 53BP1 DSB response by examining the MPL response kinetics of mCherry-F53BP1 in *WHSC1* ^−/−^ (here termed *WHSC1* ^mut/mut^) fibroblasts vs. wild type controls [45]. Surprisingly, 53BP1 response kinetics were identical in the two cultures (**Figure 6A**; **Figure S3**; **Movie S7**). Similarly, endogenous 53BP1 IR-induced focus formation was unperturbed in *WHSC1* ^mut/mut^ MEFs, even at early time points (**Figure 6B**; **Figure S4**). Western blotting for WHSC1 in *WHSC1* ^mut/mut^ MEFs revealed an off-size band, likely corresponding to an N-terminal (catalytically dead) fragment of the WHSC1 protein (**Figure 6C**). Interestingly, we noted a bias in favor of the H4K20me1 mark in *WHSC1* ^mut/mut^ MEFs, in comparison to control MEFs. To exclude a possible scaffolding function for the catalytically dead N-terminal WHSC1 fragment, we used siRNA to deplete WHSC1 (“siWHSC1”) and determined the impact on 53BP1 recruitment kinetics to MPL-induced DSBs. WHSC1-depleted wild type MEFs revealed no alteration in F-53BP1 response kinetics in comparison with parallel cultures that received control siLuc (**Figure 6D**), but revealed an increase in the intensity ratio (**Figure S5**; **Movies S8** and **S9**). The reasons for this effect are unclear. Endogenous 53BP1 IR-induced focus formation was not suppressed by siWHSC1 (**Figure 6E**), despite evidence of efficient siRNA-mediated depletion of WHSC1 in extracts from siWHSC1-treated MEFs (**Figure 6F**). In *Suv4-20h1/h2* null MEFs, siWHSC1 had no impact on either mCherry-F53BP1 MPL responses or endogenous 53BP1 IR-induced focus formation (**Figure S5**; **Movie S10** and data not shown). These results indicate that WHSC1 is not required for 53BP1 recruitment to DSBs in primary MEFs, in which either the H4K20me2 or H4K20me1 marks are densely represented in chromatin.

## Discussion

We report here a quantitative analysis of 53BP1 DSB responses, using a new MPL system that allows real time kinetic analysis. Use of MPL to study DSB responses in mammalian cells is at present limited to a small number of studies [36], [39], [46], [47]. A major benefit of MPL is the ability to focus laser energy into a tiny volume (<1 femtoliter) within the cell, generating sublethal levels of DNA damage [36], [39], [48]. In the experiments described here, the MPL was tuned to 780 nm, generating a two-photon activation wavelength equivalent of approximately 390 nm. Although the mechanism of DSB formation by MPL is not clear, 390 nm absorption by adenine nucleotide derivatives such as nictotinamide (NADH) or flavin (FAD, FMN) could lead to the secondary generation of reactive oxygen species (ROS) intermediates [49], [50]. If so, the chemistry of MPL-induced DSB formation may resembles that of IR, which is also thought to be mediated by ROS intermediates [51]. Use of MPL in this work enabled a quantitative analysis of 53BP1 responses in cells lacking distinct histone methyltransferases. The Gompertz growth pattern manifested by 53BP1 is of particular interest. Gompertzian growth is frequently observed in solid tumors and in other biological settings where potentially exponential growth is tempered by limited resources to support that growth [52]. It will be interesting to study MPL response kinetics of other DSB response factors, many of which, we anticipate, may reveal specific kinetic behaviors different to either MDC1 or 53BP1.

We report here that 53BP1 DSB response kinetics is largely unaltered by deletion of *Suv4-20 h1/h2* and the accompanying chromatin-wide conversion from H4K20me2 to H4K20me1. These results are consistent with the in vitro binding properties of the 53BP1 tandem Tudor repeat, which reveals equivalent affinities for H4K20me2 and me1, but no appreciable binding to H4K20me0 or me3 [24], [26]. In contrast, depletion of the H4K20me1 mark profoundly impaired 53BP1 recruitment to MPL-induced DSBs, as revealed by a high proportion of non-responder cells, reduced intensities of 53BP1 foci and delayed kinetics of 53BP1 recruitment. Although it seems reasonable to attribute the defective 53BP1 chromatin recruitment in cells depleted of H4K20me1 to loss of this mark, it is possible that PR-SET7 overexpression in these experiments causes additional cellular dysfunctions that contribute to the impaired 53BP1 response.

Recent work has implicated MMSET/WHSC1 in the 53BP1 response to DSBs [33] and to replication stress [44]. To further investigate the role of WHSC1 in the 53BP1 DSB response, we examined 53BP1 responses in *WHSC1* ^mut/mut^ MEFs. Surprisingly, although we did note perturbations in the balance of H4K20 methylation states in these cells,endogenous 53BP1 formed normal IR-induced foci in *WHSC1* ^mut/mut^ MEFs and the kinetics of the F53BP1 response to MPL-induced DSBs was unaltered. Similarly, acute siRNA-mediated depletion of WHSC1 in either wild type or *Suv4-20h1/2* null MEFs had no impact on endogenous 53BP1 IR-induced focus formation or on F53BP1 response kinetics to MPL-induced damage. Taken together, our results do not support a general requirement for *WHSC1* in 53BP1 DSB responses and suggest that H4K20 dimethylation is not required for the 53BP1 DSB response, provided an abundant chromatin-wide H4K20me1 or H4K20me2 mark is present. The differences between our results and those of Pei et al [33], who analyzed human cell lines, might reflect species differences. Alternatively, cell types at different stages of differentiation might exhibit different levels of dependency on specific histone methyltransferases in the regulation of 53BP1 chromatin recruitment to DSBs.

## Materials and Methods

### Plasmids

The sister chromatid recombination reporter used was described previously [34], as were **e**xpression plasmids for F53BP1 and I-SceI [19]. Retroviral vectors for *PR-Set7* expression were also described previously [26]. New constructs described here were generated by standard cloning procedures.

### Cell Lines and Cell Culture

Wild type and *Suv4-20h1/2* null immortalized MEFs were described previously [30]. Cells were maintained on gelatinized plates in DMEM supplemented with 10% fetal bovin serum (Atlanta Biologicals, Lawrenceville, GA, USA), 0.1 mM β-Mercaptoethanol (Sigma, St. Louis, MO, USA), 2 mM L-Glutamine (Mediatech, Manassas, VA, USA), 100 U penicillin/100 µg streptomycin (Gibco, Grand Island, NY, USA) and 1× MEM nonessential amino acids (Mediatech) at 37°C and 6% CO_2_. To generate HR reporter stable lines, 9 µg of KpnI-linearized HR reporter plasmid was electroporated into 9×10^6^ wild type or *Suv4-20h1/2* null cells in a 0.4 cm-electrode gap cuvette (BioRad Gene Pulser, Hercules, CA, USA, 960 mF/250V,). 0.8 mg/mL G418 (Sigma) was added to the medium 1 day after electroporation. Beginning 1 week after continuous selection, G418-resistant colonies were isolated and screened by Southern blotting for single-copy HR reporter integration.

### RNAi

Control RNAi duplex against luciferase (5′-CGUACGCGGAAUACUUCGAdTdT-3′) and RNAi SMARTpool against mouse *RNF8* and mouse *WHSC1* were purchased from Dharmacon (Lafayette, CO, USA). For siRNA knockdown 0.6×10^5^ trypsinized cells were transfected with 40 pmol siRNA using 1.92 µL Lipofectamine™ 2000 (Invitrogen, Grand Island, NY, USA) in a 24-well plate. Cells were imaged for MPL-induced recruitment of F53BP1 48 hours post-transfection, when siRNA knockdown should be near its peak.

### Antibodies and Immunoblotting

Cells were lysed in RIPA buffer (50 mM Tris-HCl [pH 8.0], 1.0% NP-40, 150 mM NaCl, 0.5% sodium deoxycholate, 0.1% SDS) supplemented with protease inhibitor cocktail (Roche, Indianapolis, IN, USA). Protein concentration was calculated using Bradford Reagent (Sigma). Histones were prepared by lysis of cells in modified nuclear extraction buffer (50 mM Tris-HCl [pH 8.0], 150 mM NaCl, 1 mM EDTA, 1.0% NP-40) supplemented with protease inhibitor cocktail (Roche) followed by extraction of histones in acid (0.5 M HCl, 10% glycerol). Cell lysates and histones were resolved by SDS-PAGE on NuPAGE® Novex Bis-Tris Gels (Invitrogen), transferred to nitrocellulose (Bio-Rad semi-dry transfer system, 250 mA 1 hr or 40 mA overnight), and blocked in 5% nonfat milk in 0.05% PBST (0.05% Tween 20, in PBS). Membranes were incubated with rabbit polyclonal anti-H4K20me1 1∶2000 (Cell Signaling Technology, Danvers, MA, USA), anti-H4K20me2 1∶1000 (Millipore, Billerica, MA, USA), anti-H4 pan 1∶10000 (Millipore), mouse monoclonal anti-WHSC1 antibody 29D1 1∶10,000 (AbCam, Cambridge, UK), anti-HA (12CA5) 1∶50 (Scully Lab) or mouse monoclonal anti-β actin 1∶10000 (AbCam, Cambridge, UK) overnight at 4°C. Membranes were washed in 0.05% PBST, incubated with peroxidase-conjugated goat anti-mouse (Jackson ImmunoResearch, West Grove, PA, USA) or Protein A (GE Healthcare, Waukesha, WI, USA) antibody, and exposed using high-sensitivity ECL (PerkinElmer, Waltham, MA, USA).

### Immunofluorescence Staining

Cells were plated at a density of 0.2×10^6^ cells/well on square glass coverslips in 6-well plates overnight prior to assay. Cells were treated with 3 or 5 Gy of γ-IR and allowed to recover for the desired length of time (0–60 min). For some stainings cells were fixed in 3% paraformaldehyde/2% sucrose (10 min), washed, permeabilized in Triton X-100 solution (0.5% Triton X-100, 20 mM HEPES [pH 7.4], 50 mM NaCl, 3 mM MgCl_2_, 300 mM sucrose) on ice, and washed. For other stainings cells were fixed in a 70∶30 methanol∶acetone solution at −20°C (20 min), dried at room temperature, and rehydrated with PBS (20 min). Cells from either fixation method were then incubated in primary antibody (anti-53BP1 1∶200 (Novus Biologicals, Littleton, CO, USA); anti-γH2AX 1∶500 (Millipore); anti-HA [12CA5] 1∶10) diluted in 5% goat serum, 0.5% sodium azide in PBS for 20 min at 37°C. Cells were washed and incubated in goat anti-mouse or rabbit conjugated FITC or Rhodamine secondary antibody (1∶200, Jackson ImmunoResearch) diluted in 5% goat serum, 0.5% sodium azide in PBS for 20 min at 37°C. Cells were washed, mounted on glass slides using ProLong® Gold antifade reagent with DAPI (Invitrogen), and imaged on a Zeiss microscope (Maple Grove, MN, USA).

### Recombination Assays

0.6×10^5^ trypsinized cells were transfected with 0.8 µg plasmid DNA using 1.92 µL Lipofectamine™ 2000 (Invitrogen) in a 24-well plate. For cotransfection 60% of the total DNA transfected was expression plasmid and 40% was I-SceI expression plasmid. Transfection efficiency was measured by parallel transfection of wt*GFP* expression vector at one-tenth of the total amount of plasmid DNA transfected. GFP^+^ frequencies were measured 72 hr post-treatment by flow cytometry using an FC500 (Beckman Coulter, Brea, CA, USA). Statistical analysis was performed using a two-tailed Student's t-test (unknown variance).

### Retroviral transduction of MEFs

8×10^6^ HEK293T cells were plated overnight on 10 cm dishes. The following day cells were cotransfected with 5 µg replication-incompetent helper vector pCL-Eco and 7.5 µg retroviral vector using 50 µL Lipofectamine™ 2000 (Invitrogen). Viral supernatant was collected 48 hours post-transfection, treated with 8 µg/mL polybrene, and used to infect 0.4×10^6^ MEF cells. Selection for retroviral transduction in 4 µg/mL puromycin began 24 hours after infection and continued for at least 3 days before cells were analyzed.

### MPL-induced DNA Damage and Fluorescence Data Collection

A Mai Tai® Ultrafast Ti:Sapphire Laser (Spectra-Physics, Santa Clara, CA, USA, 100 fs pulse, 80 MHz repetition rate) was introduced into a Nikon Ti microscope (Nikon, Melville, NY, USA) via a custom-built open beam optical path. The laser was spatially filtered to remove the non-transverse electromagnetic modes (non-TEM00) in order to generate a Gaussian excitation shape and then expanded to overfill the back aperture of a 60×1.4 numerical aperture objective (Nikon). A 675 nm low pass dichroic mirror (Chroma Technology Corp., Bellows Falls, VT, USA) was mounted in the microscope to reflect the laser into the objective. An average power level of 25 mW at 780 nm was used for all DNA damage experiments. The power was measured in the optical path outside of the microscope and is approximately three times higher than the power at the sample. Focus formation was monitored by accumulation of a mCherry-tagged F53BP1 via epifluorescence excitation. Cells were incubated in a HEPES-Leibovitz-15 based live cell media to permit a pH buffered environment for imaging. Each set of data collection began with the capture of two fluorescence images prior to DNA damage followed by exposure to the laser for five seconds. A time-lapse movie was collected for 30 min after DNA damage induction with a frame interval of 10 or 30 seconds. Data movies were analyzed in MATLAB® (The MathWorks, Natick, MA, USA) with a custom algorithm to follow focus movement, quantify intensity and generate a plot of fluorescence accumulation over time. Fitting of the fluorescence time courses to the Gompertz function were carried out using a non-linear least squares method in MATLAB to yield “intensity ratio”, “lag-time” and “slope” parameters. Intensity ratios were calculated from the raw data (without fitting) by averaging the final focus intensity (over five frames) and subtracting the background fluorescence and then dividing by pre-laser pulse intensity after background correction. The p-values quoted in the figure legends are calculated from two-tailed unpaired t-tests, unless otherwise stated. Linear fit analysis was carried out using MATLAB to calculate the Pearson correlation coefficient. For each fit, the r-squared (square of the Pearson coefficient) value is stated.

## Supporting Information

Figure S1 **Reciprocal relationship between slope and lag-time in 53BP1 response to MPL-induced DSBs.** Figure summarizes correlation analysis of lag-time *vs*. 1/slope for all cell types used in the MPL experiments. Note reduced correlation between lag-time and 1/slope in *Suv4-20h1/2* null MEFs (including those overexpressing PR-SET7) and in wild type MEFs lacking RNF8. The loss of correlation can be seen both in the graphs and the reduced r-squared (Pearson) coefficient.
(TIF)

Figure S2 **Impact of PR-Set7 expression on recruitment kinetics of MDC1 to MPL induced DSBs.** **A**)–**C**) Comparison of *Suv4-20h1/2* null (h1/2 null) MEFs expressing control vs. PR-SET7 retrovirus. **A**) Plot of intensity ratio for each responding cell. Bar represents the mean for each data set and error bars indicate SEM (p>0.3). **B**) Plot of averaged MDC1 fluorescence accumulation over time, normalized to a peak fluorescence intensity of 1.0 for each responding cell. Error bars indicate STD. **C**) Plot of MDC1 fluorescence accumulation rate for each cell imaged, with fitting by single exponential function y = a-b*exp(t/τ). τ = time taken for signal to decay by 1/*e* (related to half-life of exponential function). Bar represents the mean rate and error bars indicate SEM (p>0.4).
(TIF)

Figure S3 **Kinetic parameters of F-53BP1 MPL responses in** ***WHSC1*** **mutant MEFs.** **A**) Plot of intensity ratio for each responding wild type (WT; black circles) and *WHSC1^mut/mut^* (Whsc1 mut; red squares) cell. Bar represents the mean peak fluorescence intensity for each data set and error bars indicate SEM (p = 0.7). **B**) Plot of slope in fluorescence accumulation at the inflection point for each responding cell. Bar represents the mean slope in fluorescence accumulation at the inflection point for each data set and error bars indicate SEM (p = 0.44). **C**) Plot of lag-time in fluorescence accumulation for each MPL-induced DNA lesion. Bar represents the mean lag-time in fluorescence accumulation for each data set and error bars indicate SEM (p = 0.84). **D**) Plot of slope *vs*. intensity ratio for each responding cell. **E**) Plot of lag-time *vs*. intensity ratio for each responding cell. **F**) Plot of slope *vs*. lag-time in each responding cell.
(TIF)

Figure S4 **Quantitation of IR-induced focus formation in WT** ***vs. WHSC1^mut/mut^*** **MEFs.** Cells received 5 Gy of IR or were mock treated, then were immunostained or γ-H2AX and 53BP1 5 (panel **A**) or 10 (panel **B**) minutes later. Total number of cells scored per sample ranged from 138 to 207.
(TIF)

Figure S5 **Impact of siRNA-mediated WHSC1 depletion on 53BP1 response kinetics in** ***Suv4-20h1/2*** **null MEFs.** **A**) Plot of averaged mCherry-F53BP1 fluorescence accumulation over time normalized to a peak fluorescence intensity of 1.0 for *Suv4-20h1/2* null MEFs transfected with siLuc or siWHSC1. Error bars indicate SD. **B**) Plot of maximum fluorescence intensity for each responding cell in this experiment and in siWHSC1-depleted wild type MEFs (data from experiment shown in **Figure 6**). Bar represents the mean intensity ratio for each data set and error bars indicate SEM (t-test of siLuc vs. siWHSC1: WT p<0.06; h1/2 null p = 0.62). **C**) Plot of slope in fluorescence accumulation at the inflection point for each responding cell. Bar represents the mean and error bars indicate SEM (t-test of siLuc vs. siWHSC1: WT p = 0.33; h1/2 null p = 0.37). **D**) Plot of lag-time for each responding cell. Bar represents the mean and error bars indicate SEM (t-test of siLuc vs. siWHSC1: WT p = 0.65; h1/2 null p = 0.65). **E**) Plot of slope *vs*. intensity ratio for each responding cell. **F**) Plot of the lag-time *vs*. intensity ratio for each responding cell. F) Plot of slope *vs*. lag-time for each responding cell.
(TIF)

Table S1 **Responses of RFP-F53BP1 to different levels of multiphoton laser power in wild type MEFs.** Mean laser power was titrated as shown and response rates were determined in cells in the absence or presence of BrdU.
(PDF)

Movie S1 **Typical time course of GFP-MDC1 accumulation at sites of MPL damage: wild type MEFs.**
(AVI)

Movie S2 **Typical time course of mCherry-F53BP1 accumulation at sites of MPL damage: siLuc-treated wild type MEFs.**
(AVI)

Movie S3 **Typical time course of mCherry-F53BP1 accumulation at sites of MPL damage: siRNF8-treated wild type MEFs.** Note delayed and diminished accumulation of MPL-induced focal accumulation of mCherry signal.
(AVI)

Movie S4 **Typical time course of mCherry-F53BP1 accumulation at sites of MPL damage:** ***Suv4-20h1/h2*** **^−/−^ MEFs.**
(AVI)

Movie S5 **Typical non-responder cell in** ***Suv4-20h1/h2*** **^−/−^ MEFs expressing PR-Set7.** Note mCherry signal but absence of MPL-induced focal accumulation.
(AVI)

Movie S6 **Typical responder cell in** ***Suv4-20h1/h2*** **^−/−^ MEFs expressing PR-Set7.** Note delayed and diminished accumulation of MPL-induced focal accumulation of mCherry signal.
(AVI)

Movie S7 **Typical time course of mCherry-F53BP1 accumulation at sites of MPL damage:** ***WHSC1*** **^mut/mut^ MEFs.**
(AVI)

Movie S8 **Typical time course of mCherry-F53BP1 accumulation at sites of MPL damage: siWHSC1-treated wild type MEFs.**
(AVI)

Movie S9 **Typical time course of mCherry-F53BP1 accumulation at sites of MPL damage: siLuc-treated control wild type MEFs in same experiment as shown in Movie S8.**
(AVI)

Movie S10 **Typical time course of mCherry-F53BP1 accumulation at sites of MPL damage: siWHSC1-treated** ***Suv4-20h1/h2*** **^−/−^ MEFs.**
(AVI)
