# Supplementary figures and images for: Impact of Histone H4 Lysine 20 Methylation on 53BP1 Responses to Chromosomal Double Strand Breaks
Source: PLoS One. 2012 Nov 28;7(11):e49211. doi: 10.1371/journal.pone.0049211 (PMC3509127; doi:10.1371/journal.pone.0049211)

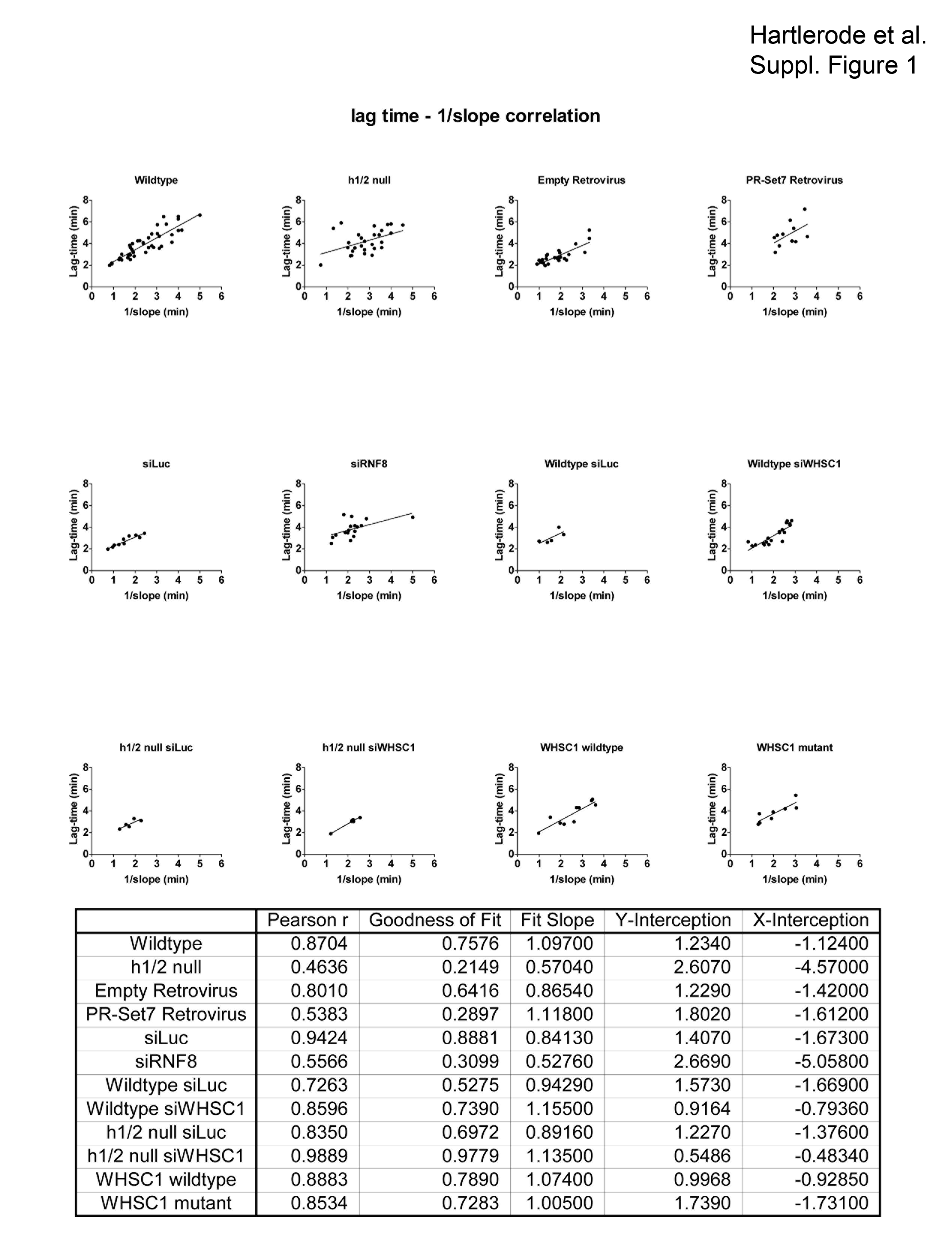

Supplement: Figure S1 — Reciprocal relationship between slope and lag-time in 53BP1 response to MPL-induced DSBs. Figure summarizes correlation analysis of lag-time vs. 1/slope for all cell types used in the MPL experiments. Note reduced correlation between lag-time and 1/slope in Suv4-20h1/2 null MEFs (including those overexpressing PR-SET7) and in wild type MEFs lacking RNF8. The loss of correlation can be seen both in the graphs and the reduced r-squared (Pearson) coefficient. (TIF) [file pone.0049211.s001.tif]

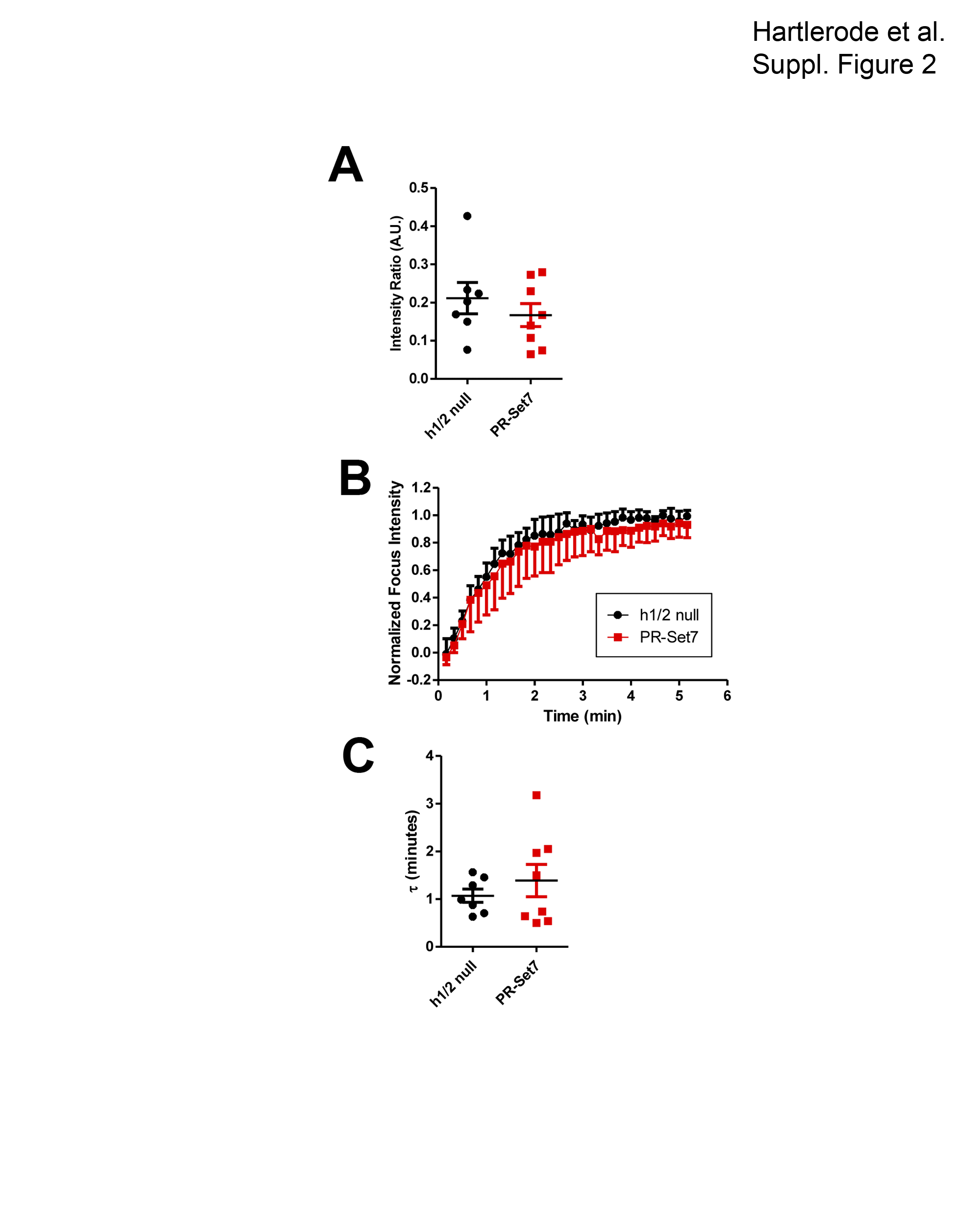

Supplement: Figure S2 — Impact of PR-Set7 expression on recruitment kinetics of MDC1 to MPL induced DSBs. A)–C) Comparison of Suv4-20h1/2 null (h1/2 null) MEFs expressing control vs. PR-SET7 retrovirus. A) Plot of intensity ratio for each responding cell. Bar represents the mean for each data set and error bars indicate SEM (p>0.3). B) Plot of averaged MDC1 fluorescence accumulation over time, normalized to a peak fluorescence intensity of 1.0 for each responding cell. Error bars indicate STD. C) Plot of MDC1 fluorescence accumulation rate for each cell imaged, with fitting by single exponential function y = a-b*exp(t/τ). τ = time taken for signal to decay by 1/e (related to half-life of exponential function). Bar represents the mean rate and error bars indicate SEM (p>0.4). (TIF) [file pone.0049211.s002.tif]

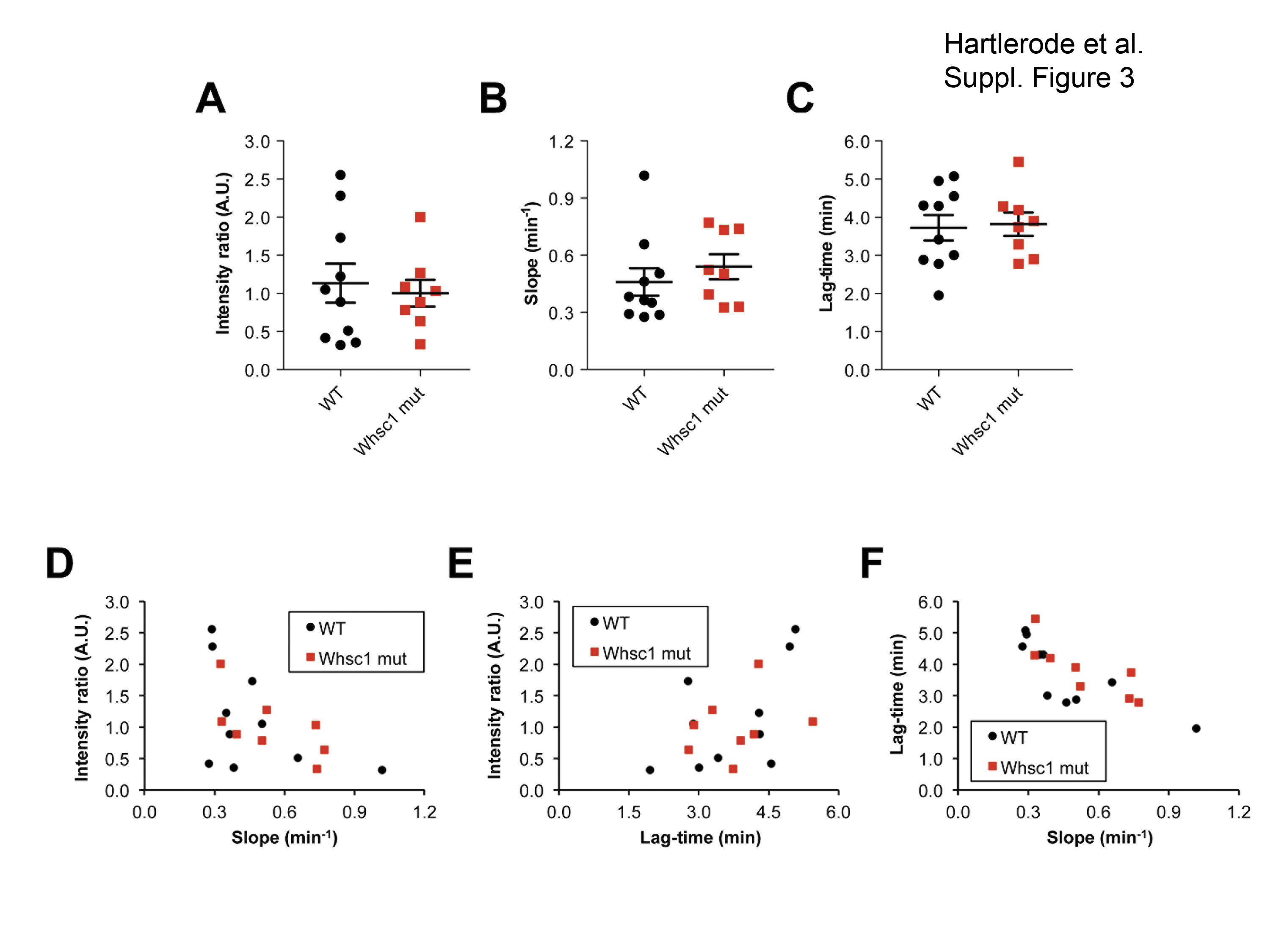

Supplement: Figure S3 — Kinetic parameters of F-53BP1 MPL responses in WHSC1 mutant MEFs. A) Plot of intensity ratio for each responding wild type (WT; black circles) and WHSC1mut/mut (Whsc1 mut; red squares) cell. Bar represents the mean peak fluorescence intensity for each data set and error bars indicate SEM (p = 0.7). B) Plot of slope in fluorescence accumulation at the inflection point for each responding cell. Bar represents the mean slope in fluorescence accumulation at the inflection point for each data set and error bars indicate SEM (p = 0.44). C) Plot of lag-time in fluorescence accumulation for each MPL-induced DNA lesion. Bar represents the mean lag-time in fluorescence accumulation for each data set and error bars indicate SEM (p = 0.84). D) Plot of slope vs. intensity ratio for each responding cell. E) Plot of lag-time vs. intensity ratio for each responding cell. F) Plot of slope vs. lag-time in each responding cell. (TIF) [file pone.0049211.s003.tif]

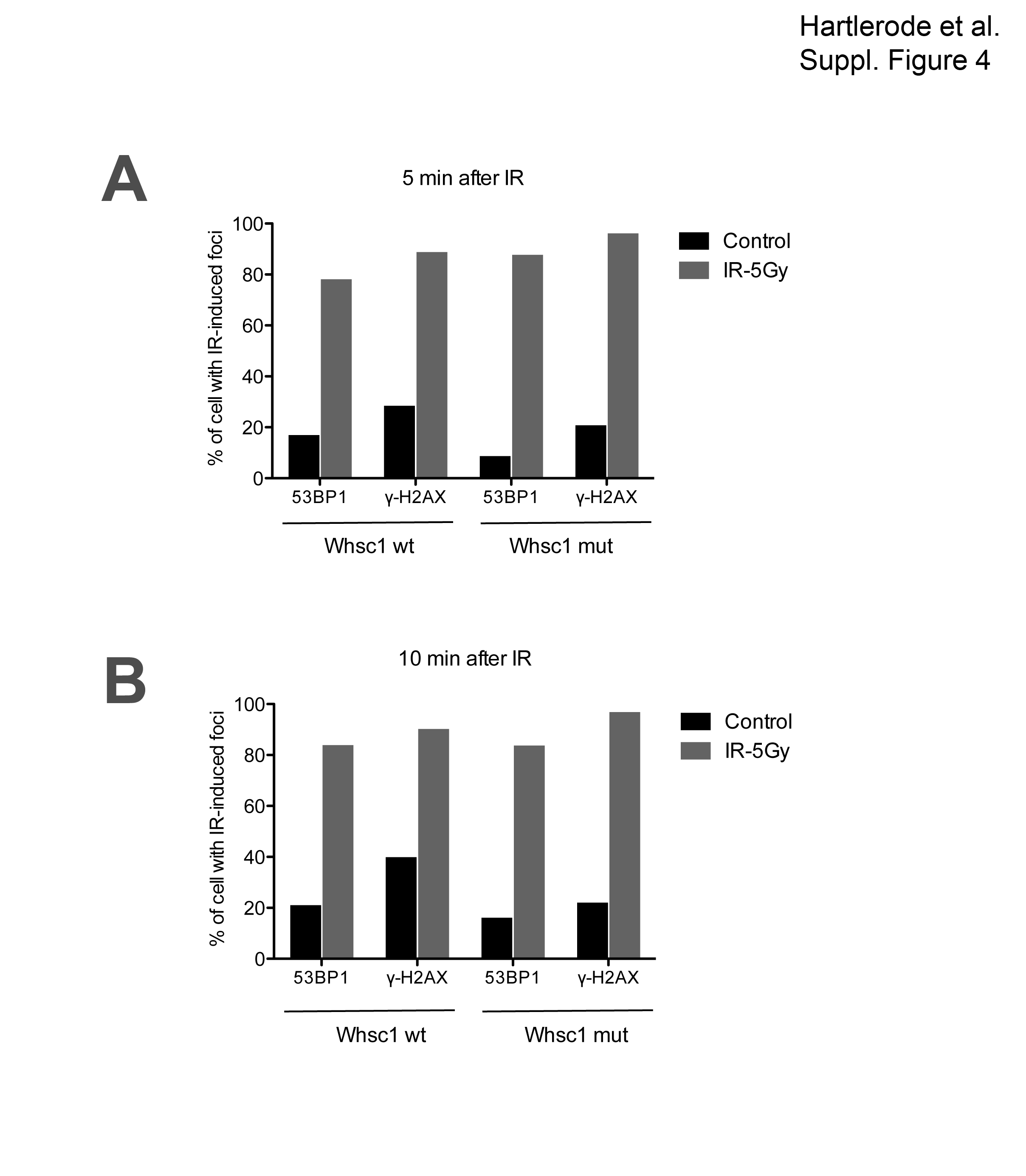

Supplement: Figure S4 — Quantitation of IR-induced focus formation in WT vs. WHSC1mut/mut MEFs. Cells received 5 Gy of IR or were mock treated, then were immunostained or γ-H2AX and 53BP1 5 (panel A) or 10 (panel B) minutes later. Total number of cells scored per sample ranged from 138 to 207. (TIF) [file pone.0049211.s004.tif]

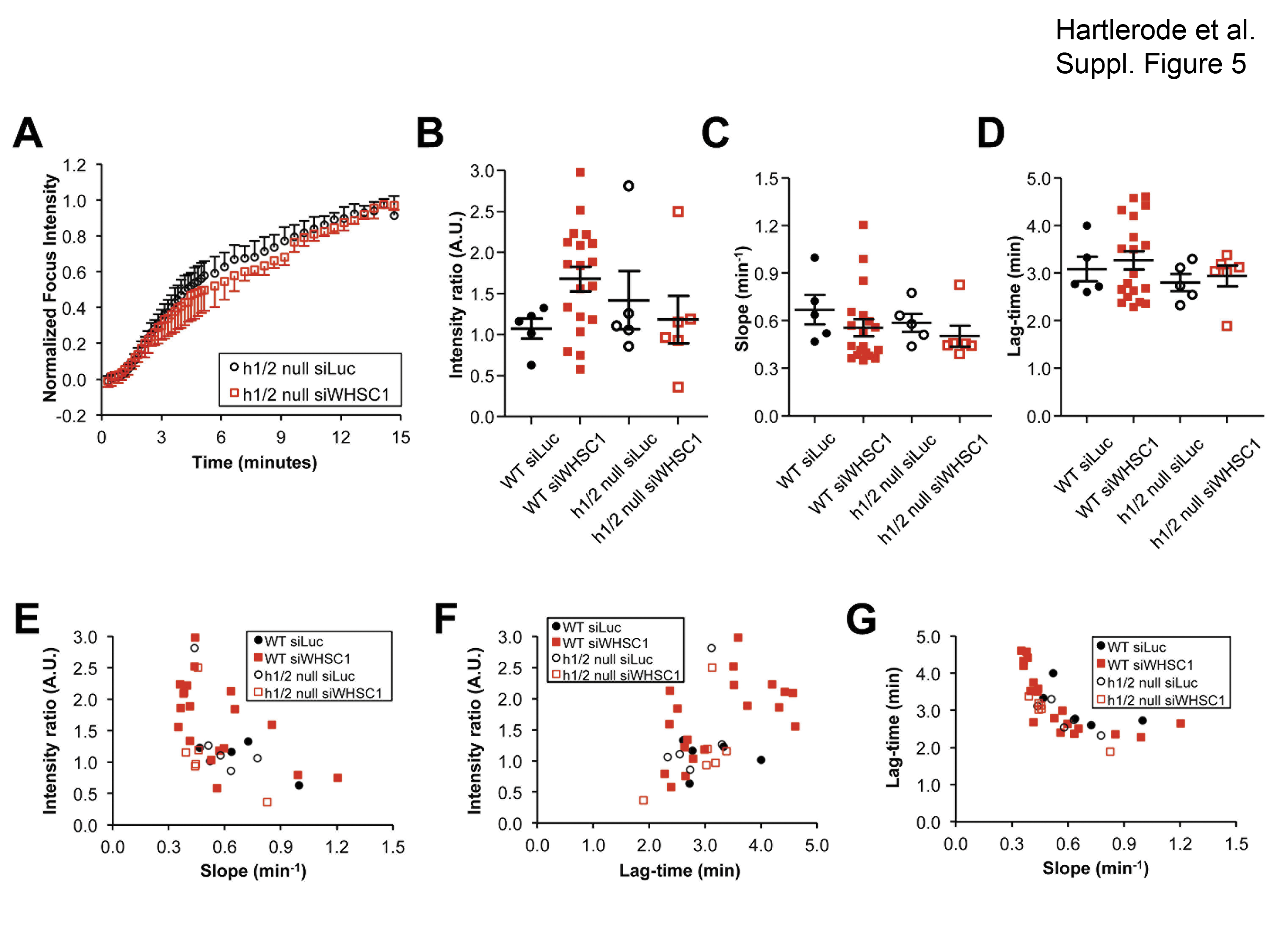

Supplement: Figure S5 — Impact of siRNA-mediated WHSC1 depletion on 53BP1 response kinetics in Suv4-20h1/2 null MEFs. A) Plot of averaged mCherry-F53BP1 fluorescence accumulation over time normalized to a peak fluorescence intensity of 1.0 for Suv4-20h1/2 null MEFs transfected with siLuc or siWHSC1. Error bars indicate SD. B) Plot of maximum fluorescence intensity for each responding cell in this experiment and in siWHSC1-depleted wild type MEFs (data from experiment shown in Figure 6 ). Bar represents the mean intensity ratio for each data set and error bars indicate SEM (t-test of siLuc vs. siWHSC1: WT p<0.06; h1/2 null p = 0.62). C) Plot of slope in fluorescence accumulation at the inflection point for each responding cell. Bar represents the mean and error bars indicate SEM (t-test of siLuc vs. siWHSC1: WT p = 0.33; h1/2 null p = 0.37). D) Plot of lag-time for each responding cell. Bar represents the mean and error bars indicate SEM (t-test of siLuc vs. siWHSC1: WT p = 0.65; h1/2 null p = 0.65). E) Plot of slope vs. intensity ratio for each responding cell. F) Plot of the lag-time vs. intensity ratio for each responding cell. F) Plot of slope vs. lag-time for each responding cell. (TIF) [file pone.0049211.s005.tif]
