# Supplementary material for: Impact of Histone H4 Lysine 20 Methylation on 53BP1 Responses to Chromosomal Double Strand Breaks
Source: PLoS One. 2012 Nov 28;7(11):e49211. doi: 10.1371/journal.pone.0049211 (PMC3509127; doi:10.1371/journal.pone.0049211)
Supplement: Table S1 — Responses of RFP-F53BP1 to different levels of multiphoton laser power in wild type MEFs. Mean laser power was titrated as shown and response rates were determined in cells in the absence or presence of BrdU. (PDF) [file pone.0049211.s006.pdf]

Hartlerode et al., Table S1.

Cell type	Mean laser power (mW)	Response
No BrdU	10	0/5
	15	4/5
	20	5/5
	25	4/5
	50	4/5
+ BrdU	2	0/3
	5	3/3
	10	3/3
	20	3/3
